# Impact of the COVID-19 Pandemic on Productivity of Workers in the Health Sector between Working in a Hospital and from Home

**DOI:** 10.3390/jcm12155129

**Published:** 2023-08-04

**Authors:** Robert M. Trojak, Melanie Lenger, Armin Birner, Alexander Maget, Nina Dalkner, Jorgos N. Lang, Frederike T. Fellendorf, Michaela Ratzenhofer, Elena M. D. Schönthaler, Eva Fleischmann, Susanne A. Bengesser, Robert Queissner, Martina Platzer, Adelina Tmava-Berisha, Eva Z. Reininghaus

**Affiliations:** Clinical Division of Psychiatry and Psychotherapeutic Medicine, Medical University Graz, 8036 Graz, Austria; robert.trojak@stud.medunigraz.at (R.M.T.); armin.birner@medunigraz.at (A.B.); alexander.maget@medunigraz.at (A.M.); nina.dalkner@medunigraz.at (N.D.); jorgos.lang@edu.uni-graz.at (J.N.L.); frederike.fellendorf@medunigraz.at (F.T.F.); michaela.ratzenhofer@t-online.de (M.R.); elena.schoenthaler@medunigraz.at (E.M.D.S.); eva.fleischmann@medunigraz.at (E.F.); susanne.bengesser@medunigraz.at (S.A.B.); robert.queissner@medunigraz.at (R.Q.); martina.platzer@medunigraz.at (M.P.); adelina.tmava-berisha@medunigraz.at (A.T.-B.); eva.reininghaus@medunigraz.at (E.Z.R.)

**Keywords:** COVID-19 pandemic, healthcare worker, productivity, mental health, working from home, frontline worker in the health sector, depression

## Abstract

Background: Due to the COVID-19 pandemic, workplaces in the medical field experienced changes. Non-frontline workers in the health sector (WHS) were in many cases allowed to work from home (WFH). Changes in work locations have affected the perception of productivity during the COVID-19 pandemic compared to the pre-pandemic perception. Studies regarding this research field are rare for WHS. The aim of the present study was to investigate the perception of productivity and its impact on symptoms of depression during the COVID-19 pandemic. The second objective was to assess the implications for post-pandemic work settings such as WFH or work scenarios in hospitals during pandemics. Methods: At three points in time during the COVID-19 pandemic (t1; *n* = 161: April 2020, t2; *n* = 1598 winter 2020/2021, t3; *n* = 1879 winter 2021/2022), an online survey of WHS (e.g., medical doctors, nurses, scientific staff) in Austria concerning their productivity in their current workplace (pre- and post-pandemic) was conducted. The online survey included questions about the perceptions of productivity changes (i.e., perceptions of lower, equal, and higher productivity, before and during the COVID-19 pandemic) in different work settings (e.g., working in a hospital or working from home), as well as standardized questionnaires like the Patient Health Questionnaire (PHQ-9), assessing symptoms of depression in WHS. Results: χ^2^ tests showed that WHS working in hospitals experienced significantly fewer fluctuations in their perceptions of productivity than WHS working from home. An analysis of variance (ANOVA) indicated that WHS with a lower perception of productivity tended to have higher self-assessed depressive symptoms. Conclusion: The possibility of remaining working in the hospital in stressful scenarios like the COVID-19 pandemic might stabilize the feeling of productivity. Moreover, productivity is associated with self-assessed depressive symptoms. Hence, looking into the reasons behind this discrepancy between WHS in hospitals and those working from home might help to improve the home office modality and to create better structures, which are related to symptoms of depression.

## 1. Introduction

The COVID-19 pandemic is a global health crisis and a great challenge to our health system, as well as to almost the whole population of the entire world [1]. Postponements of surgeries, strict regulations on examinations and hospital stays, social distancing, insecurity in general, and numerous individual restrictions burdened a large part of the world’s population from 2019 to 2023. Originating in December 2019 in Wuhan (a city in Hubei Province, China), this virus quickly spread all around the world, affecting almost every country [2]. The WHO (World Health Organization) declared the spread of severe acute respiratory syndrome coronavirus 2 (SARS-CoV-2) to be a pandemic on 11 March 2020 [2]. In addition, many healthcare workers were affected by changes in their work location. During the pandemic, most healthcare workers around the world were assigned to other departments or areas in hospitals or clinics to treat the high number of COVID-19 patients [1]. This job insecurity during the COVID-19 pandemic caused many healthcare workers to experience stress [3]. In addition, frontline (i.e., in direct contact with COVID-19 patients) workers in the health sector (WHS) seemed to experience even higher stress levels while working in hospitals during the pandemic [3,4,5]. In addition to higher stress levels, direct contact with COVID-19 patients has a negative impact on anxiety [6], depression, and insomnia [7], and quality of life was reported to decrease due to these factors [8]. Mental health problems were shown to be more pronounced in WHS due to working directly with infected patients [9].

There have been a few studies focusing on productivity (the amount of work-related output, compared to the tasks at hand) and motivation during the COVID-19 pandemic [10]. For example, in Italy, 51 administrative officers who moved to WFH at the beginning of the COVID-19 pandemic were asked to self-assess their productivity. The results of this study revealed a decrease in productivity in 39.2% and an increase in productivity in 29.4% of participants. The authors suggested that the decrease in productivity could be explained by the presence of distractions in the domestic environment and impaired interactions with colleagues. Workers who reported higher productivity might have had reduced stress and/or reduced communication times [11]. The diversity of the abovementioned results indicates that self-assessed productivity varies not only between but also within the different sectors of work.

In addition to changing work location within the hospital, working from home (WFH) was also an opportunity for several WHS. In some countries, staff who were not involved in direct care were asked to WFH. Due to the global COVID-19 pandemic, WHS were at a substantially increased risk of becoming infected with SARS-CoV-2 [1]. To prevent high spread of the infection, some WHS were given the opportunity to WFH, such as WHS who could provide telemedicine services from home or WHS who perform scientific work in addition to their clinical care. Reducing those risk factors and their negative consequences by changing the workplace to WFH offered new possibilities to both employees and employers in healthcare. In addition to new opportunities, the change of work location to WFH was also found to be a challenge for employees. For example, the perception of productivity, which displays how productive one feels in one’s current workplace, was found to be altered when compared to the productivity before the COVID-19 pandemic, depending on the field of work [10]. A Japanese study investigated the productivity changes of four manufacturing companies during the shift to WFH. They found that workers who engaged in WFH experienced more declining productivity than those who did not work from home. They reported that the main reasons for the differences were poor WFH setups and communication difficulties [12].

An American study found that, for those who had previously been working in offices and transitioned to WFH, the perception of productivity was shown to remain equal during the COVID-19 pandemic in comparison to the perception of productivity before the COVID-19 pandemic. Female, older, and high-income workers were likely to report increased productivity [10]. Another American study found that employees perceived WFH as having a strong and positive impact on their productivity and creativity at work. Productivity and creativity are enhanced when individuals identify their work and their organizations with deeper meaning in their lives and yet are still able to maintain boundaries between work and the non-work aspects of their lives [13]. By this interpretation, productivity is supported best when work is close but not too close [13]. Furthermore, an Austrian study investigating WHS found that more WHS (30.2%: men, 27.4%; women, 33.0%) felt less productive throughout the COVID-19 pandemic when WFH, whereas 12.7% (men, 13.4%; women, 11.9%) of the WHS reported higher productivity. WHS reporting decreased productivity were more frequently younger. Participants with a higher educational status reported improved productivity more frequently than those with less education [14]. Another finding in the healthcare system reported that younger WHS felt very little decrease in their productivity. About 60% of the participants strongly disagreed that WFH interfered with their ability to complete work-related tasks. Only 4.5% of the participants reported interferences due to the switch to WFH [15]. Compared to that, the focus of a similar study was to find gender disparities among researchers in the field of natural sciences. Women’s self-reported first/corresponding author’s and coauthor’s article submissions decreased significantly between the two time periods; men’s productivity metrics did not change [16]. This occupational group was researched the most regarding WFH.

Both perceptions of productivity and symptoms of depression are associated with demographic aspects like sex and age. For example, research suggests that females suffer slightly more from mental health problems like stress, depression, anxiety, and insomnia [17]. Furthermore, research also suggests that females reported additional burdens in household work [18]. This is in line with the abovementioned finding on academic female researchers, who were found to have published less than their male colleagues during the pandemic [16]. Females also reported a substantial decrease in time devoted to research [14]. Furthermore, research on age differences compared to self-assessed productivity of WFH workers appears to be relatively rare. Awada et al. [10] found higher productivity levels among older workers. Mental health problems seemed to be less prevalent in older individuals in this context [14,19,20]. Moreover, research suggests that job satisfaction decreased with increasing age in the COVID-19 pandemic [21], which might have also decreased self-assessed productivity [22].

The purpose of this study was to determine how the COVID-19 pandemic affected the subjective perceived productivity of healthcare workers over the entire pandemic period, as a function of gender and age. In addition, the impact of the work environment (i.e., home office and hospital work) and the impact of perceived productivity on symptoms of depression were examined. In the present study, we investigated whether the perception of productivity was linked with changes in symptoms of depression in WHS over the course of the COVID-19 pandemic. As the perception of productivity is impacted by the location of work and the changes due to the COVID-19 pandemic [23,24], the current study investigated whether the perception of productivity (lower vs. equal. vs. higher productivity than before the COVID-19 pandemic) differed between workplace locations (WFH vs. working in hospitals). We hypothesized that a change in the perception of productivity might have a negative effect on symptoms of depression. We hypothesized that these effects would be even more dominant over a longer period during the COVID-19 pandemic. Since longitudinal studies are rare for this topic, the present study also focuses on that aspect. According to the recent literature, we hypothesized that WFH would have a more negative impact on the perception of productivity than working in a hospital or comparable healthcare facilities. Furthermore, we hypothesized that the COVID-19 pandemic would have effects on the perceptions of productivity in WHS, depending on sex, age, and work environment. Moreover, we hypothesized that direct contact with COVID-19 patients at the hospital as a working environment would have a more pronounced impact on the perception of productivity than indirect contact with COVID-19 patients [23].

Examining these issues could help in designing prevention strategies to maintain the productivity levels and mental wellbeing of those WHS who engage in WFH, especially since this work modality seems to be persisting beyond the COVID-19 pandemic and will be part of future work in the healthcare sector. 

## 2. Materials and Methods

### 2.1. Sample Description

The methods of this study were based on an online survey (using limesurvey.org) that was sent out to WHS in different Austrian hospitals and comparable healthcare facilities, like foster homes or rehabilitation facilities, at three different points in time (t1, t2, and t3). We included all facilities in Austria, with the request to send the questionnaires internally to the employees who treat patients in a clinical setting and provide a multi-professional service (medical, nursing, physiotherapeutic, psychological, etc.). The participants were informed about the study via internal mailings from the works councils or clinical management. Participation in the online questionnaire was then voluntary. We included all participants between the ages of 18 and 70 who reported active employment at a healthcare facility. This study was conducted in accordance with the Declaration of Helsinki and was approved by the Ethics Committee of the Medical University Graz (EK-number: 32 329 ex 19/20). The participation in our questionnaire was anonymous and voluntary. Thus, for the present research question, we could not compare the results for WHS who participated in the survey more than once. All participants consented to the anonymous use of the collected data for scientific research.

### 2.2. Study Design/Procedure

This present study was a longitudinal observational study. An online survey to investigate workers in the health sector during the COVID-19 pandemic was conducted. This study was part of a large-scale study on the impact of COVID-19 on the mental health of WHS. During the first measurement in April 2020, essential institutions like hospitals and other medical facilities remained open. Healthcare workers were offered the opportunity, if possible, to partially work from home. Non-essential shops and institutions were closed in March 2020. In the first complete lockdown in Austria, there were new rules introduced, e.g., social distancing, wearing masks, and limiting contact with people from other households. During the second measurement period in winter 2020/2021, the restrictions imposed by the government on the citizens were loosened. In the third measurement period (winter 2021/2022), Austria was in its fourth lockdown. Therefore, the present study includes estimations of productivity over two years of the COVID-19 pandemic. 

### 2.3. Materials

Workers in the health sector were asked about relevant sociodemographic data and their perceptions of their productivity via an online survey, containing self-designed questions as well as standardized questionnaires. The online survey contained the following sociodemographic items: sex, age (categorized into age groups), occupation, working hours, number of night duties in a month for certain occupations (e.g., medical doctors), number of inhabitants in the place of residence (grouped in various number brackets), living situation, inner size of the domicile, outer size of the domicile, workplace, and direct or indirect contact with COVID-19 patients. Furthermore, we compared frontline WHS (i.e., those working directly with COVID-19 patients) and non-frontline WHS (no direct contact with COVID-19 patients). The standardized psychometric measures included the Anhedonia Scale [25], Patient Health Questionnaire (PHQ-9; [26], Resilience Scale (RS13, [27]), and Pittsburgh Sleep Quality Index (PSQI; [28]). The Anhedonia Scale has a Cronbach’s alpha of 0.91 and has been validated for patients with mental disorders such as depression [25]. The RS13 has been validated by factorial analyses and has a Cronbach’s alpha of 0.85 [27]. In addition, the PSQI has a Cronbach’s alpha of 0.83, and all of its components have been validated in multiple studies examining both healthy individuals and patients with mental disorders [28].

For the present study, the PHQ-9 was used for the further analyses. The PHQ-9 contains 9 questions about symptoms of depression and is used to measure the severity of depressive symptoms through questions about suicidality, mood, cognition, appetite, and self-esteem. The PHQ-9 has been continuously validated by structural clinical interviews for diagnosis (such as the SCID: Structured Clinical Interview for DSM) and has a Cronbach’s alpha of 0.79 [26].

Additionally, self-designed COVID-19-related questions (e.g., COVID-19-related fears and productivity) were created at the beginning of the pandemic in an eye-valid manner, as there were no comparative questionnaires available at that time. Please see Table 1 for the relevant questions, translated into English. The questions in the online survey were conceptualized and presented in German for WHS in Austria.

We formulated two different questions: one about the productivity changes based on the workplace of the hospital, and the other based on the productivity changes due to WFH (see Table 1). Both questions were rated on a three-point Likert scale (“less productive”, “equally productive”, and “more productive” = 1–3, respectively). For this study, only data from the sociodemographic questionnaire, the questions regarding perceptions of productivity, and the PHQ-9 were used.

### 2.4. Statistical Analysis

For descriptive purposes, nominal and ordinal variables were described in absolute and relative quantities. Differences in the self-assessed productivity based on the nominal variables sex, age, groups, and contact with COVID-19 patients, as well as workplace, were calculated with χ^2^ tests. A one-way analysis of covariance (ANCOVA) was performed to test the differences between groups of different self-assessed productivity (low vs. equal. vs. higher productivity) and the means of symptoms of depression as measured by the PHQ-9 (controlled for age and sex). The requirements for conducting an ANCOVA were met. Data were analyzed using IBM SPSS Statistics 29 (SPSS version 26.0, IBM, Armonk, NY, USA). All hypotheses were tested at a significance level of *p* < 0.05. For our one-way analysis of variance (ANOVA), we used the depression score from the PHQ-9, where we compared the means of the PHQ-9 results of the different productivity groups (lower, equal, and higher productivity).

## 3. Results

At t1, 196 WHS started the survey, and 161 completed the whole survey, indicating that data from 161 survey participants were integrated into the analysis. In total, 62% of the participants were female. At t2, 2074 healthcare workers started the survey, and 1598 completed the whole survey. In this subsample, 82% of the participants were female. At the third timepoint (t3), 1879 people started the survey, and 1563 completed the whole survey, with 78% of the participants being female. For an overview of which healthcare workers participated in this survey, see Table 2. Additionally, please find the distribution of age in Table 3. Table 2 shows the distribution of the healthcare professions and the number of participants thereof at t1, t2, and t3, with respect to their work setting (i.e., hospital vs. working from home). Furthermore, Table 3 shows the distribution of age for all participants. 

### 3.1. Perception of Productivity Working from Home vs. Hospital

**Figure 1:**
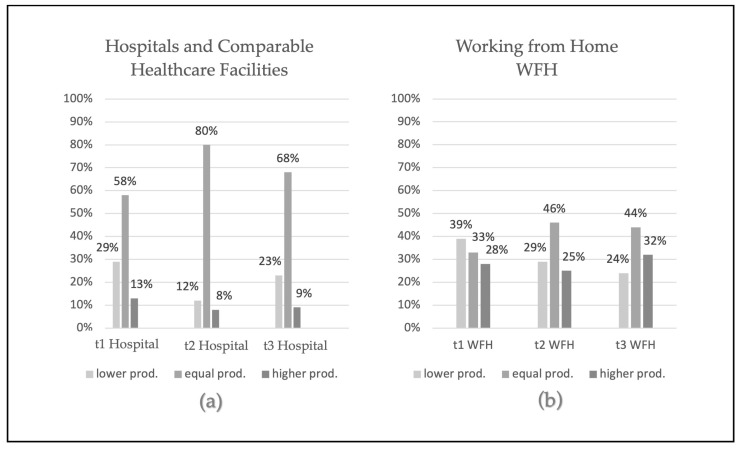
Differences in perceptions of productivity between (**a**) hospitals and (**b**) WFH in relative numbers at three different measurement times (t1, t2, and t3). Note: relative numbers of the productivity perception; prod. = productivity. The categories lower, equal, and higher indicate self-assessed productivity levels relative to the productivity before the COVID-19 pandemic.

The perception of productivity was classified into three different groups “feeling a reduction of the productivity”, “feeling an improvement in productivity”, and “feeling no changes”. See Table 4 (working in hospitals or comparable healthcare facilities) and Table 5 (WFH) for the answers of the participants at all three timepoints (Please see Figure 1 for an overview). 

Between the working locations “hospital or comparable healthcare facility” and WFH, we found a significant difference in the perceptions of productivity (during the COVID-19 pandemic). Working in hospitals and comparable healthcare facilities had a lesser effect on the perceptions of productivity than WFH at all three timepoints, where bigger differences in the perception of productivity were observed (see Table 4 and Table 5). 

### 3.2. Differences in the Perception of Productivity Depending on Age and Sex

For the age-dependent distribution in perceptions of productivity between CWP and WFH, the χ^2^ tests did not show significant age-related differences in either of the two working modalities at any of the three points in time. At the second measurement time in the working modality CWP, significant sex-dependent differences in the perceptions of productivity between males and females could be observed (see Table 6). In WHS working in a workplace at a hospital or a comparable healthcare facility, the χ^2^ tests indicated significant sex-dependent differences in the perception of productivity at the second point in time, with a significance of *p* < 0.05. The results of this analysis also showed that a higher number of female WHS (8.3%) reported higher productivity. At the same time, the number of female WHS with lower productivity was lower (10.5%; see Table 7). Individuals who engaged in WFH did not show significant sex-dependent differences in their perceptions of productivity.

### 3.3. Differences in the Perception of Productivity Depending on Whether WHS Had Direct, Indirect, or No Contact with COVID-19 Patients

The perceptions of productivity between WHS depending on whether they were directly, indirectly, or not working with COVID-19 patients did not reveal any significant differences (see Table 8).

### 3.4. Mean Comparison between Symptoms of Depression and Perceptions of Productivity

**Figure 2:**
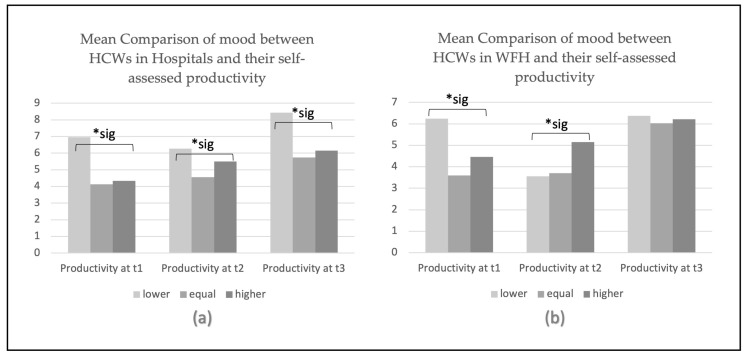
Mean comparison of symptoms of depression between WHS working in hospitals compared to WHS working from home and their perceptions of productivity. Note: the categories lower, equal, and higher indicate self-assessed productivity levels compared to the productivity before the COVID-19 pandemic; t1 = first measurement time, t2 = second measurement time, t3 = third measurement time; “WFH” = working from home; * sig = *p* < 0.05.

As shown in Table 9, we observed that an unchanged perception of productivity was associated with the lowest scores of depression, as measured by the PHQ-9 questionnaire in WHS working in the hospitals or comparable healthcare facilities. Specifically, lower numeric values of the productivity score indicated less symptoms of depression (that is, lower depression severity) in WHS. WHS working in a hospital, with the lowest (best) score for symptoms of depression, were the ones who described their productivity as being the same as it was before the pandemic, as shown in Table 9 and Table 10 and Figure 2. In contrast, WHS in hospitals with lower perceptions of productivity also had the highest (worst) scores in the PHQ-9 depression severity measurement. Similarly, WHS working in their usual workplace with higher perceptions of productivity reported lower depression scores.

WHS engaging in WFH had different results from those working in a hospital, as shown in Table 10 and Figure 2. The highest PHQ-9 depression score was found in WHS at t1, who perceived their productivity as being lower than before the pandemic. 

## 4. Discussion

Our study focused on the differences in the perceptions of productivity in WHS working at a hospital or a comparable healthcare facility versus WHS in a WFH modus, resulting in significant differences between the two workplace modalities. In the present study, we additionally analyzed the differences in perceptions of productivity based on sex and age, but we could not find significant differences based on these factors. Furthermore, we investigated whether contact with COVID-19 patients had effects on the perception of productivity, finding no significant differences. Additionally, we looked at how changes in perceptions of productivity impacted the self-rating on the severity of depression symptoms, as measured by the PHQ-9, with significant outcomes revealing that most WHS who reported a decrease in their perception of productivity also reported the highest mean PHQ-9 depression scores, indicating that an increase in symptoms of depression is more prevalent in this category. It is already known that depression and anxiety were more pronounced in WHS during the COVID-19 pandemic and resulted in lower quality of life [5,6,7,8].

In addition, we hypothesized that WFH would have a more pronounced impact on the perception of productivity than working in a common workplace, e.g., a hospital or a comparable facility; this hypothesis was supported by the results of our study. This is in line with other research projects indicating that WHS engaging in WFH were more impacted by the pandemic and felt changes in their perceptions of productivity more often than WHS working in a hospital [14].

We grouped our participants based on their perceptions of productivity, ranging from a decrease in the perception of productivity [10,15,16], to mostly no changes [10], and lastly to an increase in the perception of productivity [1,29], Overall, the data of workers who engaged in WFH do not point in just one direction [13]. This supports our first hypothesis, which posited that the shift to WFH during the COVID-19 pandemic had a more pronounced impact on the perception of productivity. Possibly, changing working modalities like the workplace initiates distress that might affect the perception of productivity. Moreover, differences in environmental factors between these two modalities might have affected productivity. For example, parents with children at home could be distracted by their presence while working [29]. In other cases, workers with increased perceptions of productivity also reported higher rates of creativity [13]. Diving deeper into the causal mechanisms and further factors of these differences would be beneficial for both WHS and employers.

Assessing whether WFH could be beneficial, as well as to whom and under which circumstances, could have a significant impact on our work life in the decision of whether one might benefit from this change, especially since WFH might be relevant in the future of our work modalities. Further data and assessment would be needed to specifically determine which groups of workers would benefit the most from WFH, as well as how we can support workers who engage in WFH to feel more productive. Regarding our data, individuals who felt highly productive and were in the WFH group also experienced an increase in symptoms of depression in line with the three timepoints. One could speculate that individuals who feel more productive while WFH should be supported to have this opportunity.

The second hypothesis, which posited that the COVID-19 pandemic had effects on the perceptions of productivity among WHS, with a dependence on sex and age, showed significant results at the second measurement time in the hospital group. Perceptions of productivity in individuals WFH did not differ based on sex and age at any of the three measurement times. These results led to the conclusion that sex had no significant impact on the perception of productivity in our data. This was not consistent with other research suggesting that there are differences in perceptions of productivity based on sex [16,24], or age [10]. Although current research shows differences between the sexes during the COVID-19 pandemic (e.g., a higher loss of productivity in female scientists [16,26] or a higher prevalence of mental health problems in females [17], which might be partially connected to more additional burdens in household work [18], our data did not show any significant differences between female and male WHS’s perceptions of productivity. The main purpose of this study was to find differences in the distribution of productivity between the sexes and age groups. 

Thirdly, we theorized that direct contact with COVID-19 patients at the hospital as a working environment would have a bigger impact on the perception of productivity than indirect or no contact with COVID-19 patients. Contrary to our assumptions, there were no significant differences in perceptions of productivity between those in direct, indirect, or no contact with COVID-19 patients. This is consistent with recent research results indicating that direct contact with COVID-19 patients has effects on mental health, e.g., stress [5], anxiety [6], depression, insomnia [7], and quality of life [8]. Deteriorated mental health could, in turn, negatively influence one’s perception of productivity. The literature indeed indicates that contact with COVID-19 patients has an effect on mental health, but comparable studies investigating the perceptions of productivity among WHS working with COVID-19 patients are rare. 

Finally, it was assumed that an improvement or decline in the perception of productivity would have a negative effect on symptoms of depression. The literature on the link between symptoms of depression and perceptions of productivity revealed a scarcity on this topic. Most studies investigated the link between general mental health and perceptions of productivity, reporting that worsening of the mental health status also increased the loss in perceptions of productivity [4,23]. As shown above, we observed that WHS working in the hospital who had an increased or decreased perceptions of productivity had worse PHQ-9 depression scores than WHS who described their productivity as being the same as it was before [24]. The worst reported PHQ-9 depression score was at the third timepoint for WHS working in the hospital who self-assessed their productivity as lower than it was before the COVID-19 pandemic. These data suggest that WHS with an increased perception of productivity still reported higher depression scores than WHS who reported equal productivity at all three measurement times at the hospital. The lowest depression scores were found in WHS who reported an unchanged perception of productivity when working at a hospital or comparable healthcare facility at all three measurement times.

### 4.1. Limitations

The current study applied self-assessment questionnaires, which limits the objectivity of the impact of perceptions of productivity on objective measurements of productivity. Furthermore, it was sent out via an online survey, and the setting was not controllable. Furthermore, the distribution of the sexes in the questionnaire revealed a dominance of female WHS in this study, which might limit the generalizability of the current study. In addition, many healthcare workers changed their usual workplace at the beginning of the pandemic because healthcare was limited to essential treatments. This may have affected the comparison of productivity before and during COVID-19. 

### 4.2. Implications

Researching the impacts of the COVID-19 pandemic on perceptions of productivity revealed differences in the perception of productivity between those working in a hospital as their usual workplace and those WFH due to the COVID-19 pandemic. Further research into the differences between these two working modalities and their benefits and downsides would be needed to broaden the knowledgebase of WFH to, on the one hand, be better prepared for similar pandemics that might occur in the future, and on the other hand, to explore new possibilities with this for many industries as a new working modality. Providing further research would be beneficial for the WHS as well as the employers. Assessing whether WFH could be beneficial, and to whom and under which circumstances, could have a big impact on our work life. The shift to WFH alters communication with other colleagues, changes the financial structures of workplaces and, thus, affects the mental health of WHS. Productivity gains from WFH would, of course, also prompt employers to consider home office options if the results of a related study confirm the benefits of WFH.

## 5. Conclusions

Our study investigated how the perceptions of productivity changed in the time of the COVID-19 pandemic between hospitals and the new workplace (WFH), with the results indicating that, indeed, WFH has larger groups of WHS perceiving their productivity as lower or higher than before the COVID-19 pandemic and the shift to WFH. Furthermore, we researched how the perceptions of productivity differed by sex and age, finding no significant differences. The present study investigated the possibility that contact with COVID-19 patients could have an impact on WHS’s perceptions of productivity, with no significant results. Furthermore, we investigated how the means of depression scores were distributed among the different groups based on their perception of productivity (lower, equal, and higher). The present study is important and necessary for the topic of WFH to gain a better understanding of this rather new working modality, its uses, and its benefits and disadvantages.

## Figures and Tables

**Table 1:** Productivity-related questions in the online survey.

Questions	Answers
How would you describe your subjective productivity now, compared to before the COVID-19 pandemic at the hospital?	(a) Equally productive (b) Less productive (c) More productive
How would you describe your subjective productivity now, compared to before the COVID-19 pandemic while WFH?	(a) Equally productive (b) Less productive (c) More productive

Note: Original questions were conceptualized in German. WFH = working from home.

**Table 2:** Professions of the healthcare workers at all three timepoints who reported productivity in hospital and WFH settings.

	Number of Participants					
Healthcare Profession	t1		t2		t3	
	Hospital	WFH	Hospital	WFH	Hospital	WFH
Medical doctors		50	103	63	101	9
Medical doctors in training		19	37	8	38	3
Psychologists and therapists		0	129	44	20	3
Medical technical assistants		2	108	16	93	5
Scientific staff		19	13	25	13	18
Nurses or nursing assistants		0	717	23	474	4
Facility management		0	15	16	16	1
Administrative staff and others		0	174	105	135	46

Note: t1 = first measurement time; t2 = second measurement time; t3 = third measurement time; WFH = healthcare workers who were working from home; Hospital = WHS working in hospitals and comparable healthcare facilities. The category “Psychologists and Therapists” includes physiotherapists, occupational therapists, psychotherapists, speech therapists, and social workers. The category “Administrative Staff and Others” includes workers who could not identify with any of the given categories.

**Table 3:** Age-dependent distribution.

	Age	18–30	31–40	41–50	51–60	61–70	71–80
t1	Hospital	13	28	16	28	4	1
	WFH	24	55	28	28	6	0
t2	Hospital	344	319	343	258	20	1
	WFH	46	82	65	81	20	1
t3	Hospital	292	246	244	195	25	1
	WFH	20	24	23	16	5	1

Note: t1 = first measurement time; t2 = second measurement time; t3 = third measurement time; WFH = healthcare workers who were working from home; Hospital = WHS working in hospitals and comparable healthcare facilities. According to the works councils of the leading hospitals in Austria, age could only be queried in ranges and not in absolute terms, due to data protection laws.

**Table 4:** Overview of the perceptions of productivity in the hospital, as measured by χ^2^ tests.

Perception of Productivity	Lower (*n*)	Equal (*n*)	Higher (*n*)	χ^2^	*p*
t1	28.9% (26)	57.8% (52)	13.3% (12)		
t2	11.7% (152)	80.3% (1041)	7.9% (103)	68.93	0.001 **
t3	22.6% (277)	68.2% (837)	9.3% (114)	df = 4	

Note: Bracketed numbers are the absolute numbers. t1 = first measurement time; t2 = second measurement time; t3 = third measurement time. The categories lower, equal, and higher indicate self-assessed productivity levels relative to the productivity before the COVID-19 pandemic; ** *p* < 0.01.

**Table 5:** Overview of the perceptions of productivity while WFH, as measured by χ^2^ tests.

Perception of Productivity	Lower (*n*)	Equal (*n*)	Higher (*n*)	χ^2^	*p*
t1	39.0% (55)	33.3% (47)	27.7% (39)		
t2	29.3% (87)	45.8% (136)	24.9% (74)	9.73	0.045 *
t3	24.6% (28)	43.9% (50)	31.6% (36)	df = 4	

Note: Bracketed numbers are the absolute numbers. t1 = first measurement time; t2 = second measurement time; t3 = third measurement time. The categories lower, equal, and higher indicate self-assessed productivity levels compared to the productivity before the COVID-19 pandemic; * *p* < 0.05.

**Table 6:** Sex-dependent distribution in perceptions of productivity in the hospital at three different measurement times (t1, t2, and t3), as measured by χ^2^ tests.

Perception of Productivity	Lower (*n*)	Equal (*n*)	Higher (*n*)	χ^2^(2)	*p*
Male t1	25.6 (10)	61.5 (24)	12.8 (5)		
Female t1	32.0 (16)	54.0 (27)	14.0 (7)	0.54	0.762
Male t2	17.6 (37)	76.2 (160)	6.2 (13)		
Female t2	10.5 (114)	81.1 (877)	8.3 (90)	9.06	0.011 *
Male t3	26.2 (66)	63.9 (161)	9.9 (25)		
Female t3	21.7 (210)	69.1 (670)	9.2 (89)	2.73	0.255

Note: numbers = relative numbers; bracketed numbers = absolute numbers; t1 = first measurement time, t2 = second measurement time, t3 = third measurement time. The categories lower, equal, and higher indicate self-assessed productivity levels compared to the productivity before the COVID-19 pandemic; * *p* < 0.05.

**Table 7:** Sex-dependent distribution in the perceptions of productivity working from home at three different measurement times (t1, t2, and t3), as measured by χ^2^ tests.

Perception of Productivity	Lower (*n*)	Equal (*n*)	Higher (*n*)	χ^2^(2)	*p*
Male t1	43.1 (22)	33.3 (17)	23.5 (12)		
Female t1	36.7 (33)	33.3 (30)	30.0 (27)	0.84	0.656
Male t2	31.6 (24)	48.7 (37)	19.7 (15)		
Female t2	28.5 (63)	44.8 (99)	26.7 (59)	1.47	0.480
Male t3	37.0 (10)	40.7 (11)	22.2 (6)		
Female t3	44.2 (38)	20.9 (18)	34.9 (30)	3.24	0.198

Note: numbers = relative numbers; bracketed numbers = absolute numbers; t1 = first measurement time, t2 = second measurement time, t3 = third measurement time. The categories lower, equal, and higher indicate self-assessed productivity levels compared to the productivity before the COVID-19 pandemic.

**Table 8:** Perceptions of productivity between direct, indirect, or no contact with COVID-19 patients.

Perception of Productivity	Lower (*n*)	Equal (*n*)	Higher (*n*)	χ^2^(4)	*p*
No contact t1	28.3 (13)	56.5 (26)	15.2 (7)		
Indirect contact t1	30.4 (7)	60.9 (14)	8.7 (2)	0.59	0.965
Direct contact t1	28.6 (6)	57.1 (12)	14.3 (3)		
No contact t2	11.1 (112)	80.9 (820)	8.0 (81)		
Indirect contact t2	14.3 (17)	78.2 (93)	7.6 (9)	2.04	0.728
Direct contact t2	14.0 (23)	78.0 (128)	7.9 (13)		
No contact t3	21.1 (126)	70.6 (422)	8.4 (50)		
Indirect contact t3	21.1 (31)	67.3 (99)	11.6 (17)	4.38	0.357
Direct contact t3	24.8 (120)	65.4 (316)	9.7 (47)		

Note: numbers = relative numbers; bracketed numbers = absolute numbers; t1 = first measurement time, t2 = second measurement time, t3 = third measurement time. The categories lower, equal, and higher indicate self-assessed productivity levels compared to the productivity before the COVID-19 pandemic. No contact, Indirect contact, and Direct contact refer to contact with COVID-19 patients.

**Table 9:** Mean comparison of symptoms of depression between WHS in hospital and their perceptions of productivity.

Measurement Time	Lower Prod.		Equal Prod.		Higher Prod.		*F*	*p*
	M	SD	M	SD	M	SD		
t1 Hospital PHQ-9	6.96	4.69	4.13	4.38	4.33	3.68	3.74	0.028 *
t2 Hospital PHQ-9	6.27	4.27	4.56	4.10	5,49	4.97	11.24	0.001 **
t3 Hospital PHQ-9	8.37	5.38	5.55	4.64	6.28	5.53	30.88	0.001 **

Note: M = mean, SD = standard deviation. Numbers in the categories lower, equal, and higher prod. indicate the mean scores of the Patient Health Questionnaire (PHQ-9) for depression; “prod.” = productivity; t1 = first measurement time, t2 = second measurement time, t3 = third measurement time. The categories lower, equal, and higher indicate self-assessed productivity levels compared to the productivity before the COVID-19 pandemic; * *p* < 0.05; ** *p* < 0.01.

**Table 10:** Mean comparison of symptoms of depression between WHS working from home and their perceptions of productivity.

Measurement Time	Lower Prod.		Equal Prod.		Higher Prod.		*F* (2)	*p*
	M	SD	M	SD	M	SD		
t1 WFH PHQ-9	6.24	5.03	3.60	3.29	4.46	3.95	5.21	0.007 **
t2 WFH PHQ-9	3.56	2.90	3.70	3.55	5.15	4.30	4.78	0.009 **
t3 WFH PHQ-9	6.37	5.23	6.02	4.73	6.22	5.500	0.04	0.959

Note: numbers in the categories lower, equal, and higher prod. indicate the mean scores of the Patient Health Questionnaire (PHQ-9) for depression; “prod.” = productivity; t1 = first measurement time, t2 = second measurement time, t3 = third measurement time. The categories lower, equal, and higher indicate self-assessed productivity levels compared to the productivity before the COVID-19 pandemic; “WFH” = working from home; ** *p* < 0.01.

## Data Availability

Not applicable.

## References

[1] World Health Organization Archived: WHO Timeline—COVID-19. https://www.who.int/news/item/27-04-2020-who-timeline---covid-19.

[2] Gómez-Ochoa S.A., Franco O.H., Rojas L.Z., Raguindin P.F., Roa-Díaz Z.M., Wyssmann B.M., Guevara S.L.R., Echeverría L.E., Glisic M., Muka T. (2021). COVID-19 in Health-Care Workers: A Living Systematic Review and Meta-Analysis of Prevalence, Risk Factors, Clinical Characteristics, and Outcomes. Am. J. Epidemiol..

[3] Billings J., Ching B.C.F., Gkofa V., Greene T., Bloomfield M. (2021). Experiences of frontline healthcare workers and their views about support during COVID-19 and previous pandemics: A systematic review and qualitative meta-synthesis. BMC Health Serv. Res..

[4] De Oliveira C., Saka M., Bone L., Jacobs R. (2022). The Role of Mental Health on Workplace Productivity: A Critical Review of the Literature. Appl. Health Econ. Health Policy.

[5] Hurst K.T., Ballard E.D., Anderson G.E., Greenstein D.K., Cavanaugh G.W., Dwyer E., Swartz K., Zarate C.A., Chung J.Y., Park L.T. (2022). The mental health impact of contact with COVID-19 patients on healthcare workers in the United States. Psychiatry Res..

[6] Biber J., Ranes B., Lawrence S., Malpani V., Trinh T.T., Cyders A., English S., Staub C.L., McCausland K.L., Kosinski M. (2022). Mental health impact on healthcare workers due to the COVID-19 pandemic: A U.S. cross-sectional survey study. J. Patient-Rep. Outcomes.

[7] De Kock J.H., Latham H.A., Leslie S.J., Grindle M., Munoz S.-A., Ellis L., Polson R., O’Malley C.M. (2021). A rapid review of the impact of COVID-19 on the mental health of healthcare workers: Implications for supporting psychological well-being. BMC Public Health.

[8] Uphoff E.P., Lombardo C., Johnston G., Weeks L., Rodgers M., Dawson S., Seymour C., Kousoulis A.A., Churchill R. (2021). Mental health among healthcare workers and other vulnerable groups during the COVID-19 pandemic and other coronavirus outbreaks: A rapid systematic review. PLoS ONE.

[9] Deo A., Mohammadnezhad M. (2022). Frontline Health Care Workers’ (HCWs) perception of barriers to managing COVID-19 in Fiji. Front. Public Health.

[10] Awada M., Lucas G., Becerik-Gerber B., Roll S. (2021). Working from home during the COVID-19 pandemic: Impact on office worker productivity and work experience. Work.

[11] Moretti A., Menna F., Aulicino M., Paoletta M., Liguori S., Iolascon G. (2020). Characterization of Home Working Population during COVID-19 Emergency: A Cross-Sectional Analysis. Int. J. Environ. Res. Public Health.

[12] Kitagawa R., Kuroda S., Okudaira H., Owan H. (2021). Working from home and productivity under the COVID-19 pandemic: Using survey data of four manufacturing firms. PLoS ONE.

[13] George T.J., Atwater L.E., Maneethai D., Madera J.M. (2022). Supporting the productivity and wellbeing of remote workers. Organ. Dyn..

[14] Weitzer J., Papantoniou K., Seidel S., Klösch G., Caniglia G., Laubichler M., Bertau M., Birmann B.M., Jäger C.C., Zenk L. (2021). Working from home, quality of life, and perceived productivity during the first 50-day COVID-19 mitigation measures in Austria: A cross-sectional study. Int. Arch. Occup. Environ. Health.

[15] Brault M.E., Laudermith A., Kroll-Desrosiers A. (2023). Telemedicine During COVID-19 Response: A Welcome Shift for Younger Female Healthcare Workers. J. Gen. Intern. Med..

[16] Krukowski R.A., Jagsi R., Cardel M.I. (2021). Academic Productivity Differences by Gender and Child Age in Science, Technology, Engineering, Mathematics, and Medicine Faculty During the COVID-19 Pandemic. J. Women’s Health.

[17] Liu S., Yang L., Zhang C., Xu Y., Cai L., Ma S., Wang Y., Cai Z., Du H., Li R. (2021). Gender differences in mental health problems of healthcare workers during the coronavirus disease 2019 outbreak. J. Psychiatr. Res..

[18] Mele B.S., Holroyd-Leduc J.M., Harasym P., Dumanski S.M., Fiest K., Graham I.D., Nerenberg K., Norris C., Parsons Leigh J., Pilote L. (2021). Healthcare workers’ perception of gender and work roles during the COVID-19 pandemic: A mixed-methods study. BMJ Open.

[19] Bruine de Bruin W. (2021). Age Differences in COVID-19 Risk Perceptions and Mental Health: Evidence from a National U.S. Survey Conducted in March 2020. J. Gerontol. Ser. B.

[20] Schou-Bredal I., Bonsaksen T., Ekeberg Ø., Skogstad L., Grimholt T.K., Heir T. (2022). A comparison between healthcare workers and non-healthcare workers’ anxiety, depression and PTSD during the initial COVID -19 lockdown. Public Health Pract..

[21] Makowicz D., Lisowicz K., Bryniarski K., Dziubaszewska R., Makowicz N., Dobrowolska B. (2022). The impact of the COVID-19 pandemic on job satisfaction among professionally active nurses in five European countries. Front. Public Health.

[22] Martin L., Hauret L., Fuhrer C. (2022). Digitally transformed home office impacts on job satisfaction, job stress and job productivity. COVID-19 findings. PLoS ONE.

[23] Yaghoubi M., Salimi M., Meskarpour-Amiri M. (2022). Systematic review of productivity loss among healthcare workers due to Covid-19. Int. J. Health Plan. Manag..

[24] Heo S., Peralta P.D., Jin L., Pereira Nunes C.R., Bell M.L. (2022). Differences in self-perception of productivity and mental health among the STEMM-field scientists during the COVID-19 pandemic by sex and status as a parent: A survey in six languages. PLoS ONE.

[25] Franz M., Meyer T., Ehlers F., Runzheirmer P., Gallhofer B. (1998). German version of the Snaith-Hamilton-Pleasure Scale (SHAPS-D): Assessing anhedonia in schizophrenic patients. Eur. Psychiatry.

[26] Gräfe K., Zipfel S., Herzog W., Löwe B. (2004). Screening psychischer Störungen mit dem “Gesundheitsfragebogen für Patienten (PHQ-D)”. Diagnostica.

[27] Leppert K., Koch B., Brähler E., Strauß B. (2008). Die Resilienzskala (RS)–Überprüfung der Langform RS-25 und einer Kurzform RS-13. Klin. Diagn. Und Eval..

[28] Buysse D.J., Reynolds C.F., Monk T.H., Berman S.R., Kupfer D.J. (1989). The Pittsburgh Sleep Quality Index: A new instrument for psychiatric practice and research. Psychiatry Res..

[29] Ipsen C., van Veldhoven M., Kirchner K., Hansen J.P. (2021). Six Key Advantages and Disadvantages of Working from Home in Europe during COVID-19. Int. J. Environ. Res. Public Health.

